# Long-Term Effects of Elexacaftor/Tezacaftor/Ivacaftor on Nocturnal Cardiorespiratory Polygraphy Parameters in Patients with Cystic Fibrosis: A Prospective Study

**DOI:** 10.3390/life15121942

**Published:** 2025-12-18

**Authors:** Monica Tosto, Giuseppe Fabio Parisi, Santiago Presti, Maria Papale, Giulia Pecora, Enza Mulè, Vittorio Ornato, Donatella Aloisio, Sara Manti, Salvatore Leonardi

**Affiliations:** 1 Pediatric Respiratory Unit, Department of Clinical and Experimental Medicine, San Marco Hospital, University of Catania, Via Carlo Azeglio Ciampi, 95121 Catania, Italyleonardi@unict.it (S.L.); 2 Department of Human Pathology in Adult and Developmental Age “Gaetano Barresi”, University of Messina, Via Consolare Valeria 1, 98124 Messina, Italy

**Keywords:** cystic fibrosis, elexacaftor/tezacaftor/ivacaftor, sleep respiratory disorders, lung function, cardiorespiratory polygraphy

## Abstract

Cystic fibrosis (CF) is a genetic disorder caused by mutations in the CFTR gene, leading to multi-system impairment. Sleep respiratory disorders (SRDs) are frequent in individuals with CF—even in those with normal or mildly impaired lung function—and may adversely affect overall health. The triple combination of elexacaftor, tezacaftor, and ivacaftor (ETI) has markedly improved clinical outcomes in CF; however, its long-term impact on SRDs remains unclear. This study aimed to assess the effects of ETI on nocturnal cardiorespiratory parameters in individuals with CF over a two-year period. Thirty-five clinically stable patients aged ≥13 years, eligible for ETI therapy, were enrolled. Nocturnal cardiorespiratory polygraphy and spirometry were performed at baseline (T0), one year (T1), and two years (T2) after ETI initiation. After one year, significant improvements were observed in mean oxygen saturation (mSpO_2_), time with SpO_2_ ≤ 90% (t ≤ 90%), and respiratory rate. Spirometric indices (FEV_1_, FVC, FEF) also significantly increased (*p* < 0.05). Correlation analysis revealed positive associations between mSpO_2_ and FEV_1_ (ρ = 0.515, *p* = 0.002) and between FEV_1_ and FVC (ρ = 0.894, *p* < 0.001), while t ≤ 90% negatively correlated with FEV_1_ (ρ = −0.404, *p* = 0.016). No additional significant changes were found at T2. ETI therapy resulted in sustained improvements in nocturnal oxygenation and lung function, supporting the importance of nocturnal respiratory monitoring during follow-up.

## 1. Introduction

Cystic fibrosis (CF) is an autosomal recessive genetic disorder caused by variants in the cystic fibrosis transmembrane conductance regulator (CFTR) gene, which encodes an epithelial ion channel responsible for chloride and bicarbonate transport across cell membranes [1]. Mutations in this gene impair ion exchange, leading to thickened secretions, chronic respiratory infections, pancreatic insufficiency, and progressive lung disease. In recent years, CF care has advanced markedly with the introduction of CFTR modulators that enhance protein function by improving chloride ion transport or promoting correct protein folding and trafficking to the cell membrane [2,3]. A highly effective CFTR modulator, the triple combination of elexacaftor, tezacaftor, and ivacaftor (ETI), is approved for individuals with at least one F508del mutation and widely prescribed to patients aged 6 and older [2,4]. ETI has shown exceptional clinical effectiveness, altering disease progression with improvements in lung function, fewer pulmonary exacerbations, lower sweat chloride levels, reduced hospitalizations, higher body mass index, enhanced quality of life, and decreased infections and mortality. Furthermore, ETI has shown a favorable safety and tolerability profile [5,6,7,8,9,10,11]. However, the impact of ETI on sleep respiratory disorders (SRDs) has yet to be thoroughly evaluated.

SRDs are a significant yet often overlooked comorbidity in people with CF, even in those who are clinically stable and have normal to mildly impaired lung function [12,13,14,15]. These include obstructive sleep apnea, nocturnal hypoxemia, and nocturnal hypoventilation, which may arise from chronic lung disease, nocturnal cough, sinusitis, obesity, and gastrointestinal disorders [16,17,18]. These disturbances often arise early and can worsen pulmonary, cardiovascular, metabolic, and cognitive health, underscoring the need for effective screening and treatment strategies [17]. Interest in sleep studies for people with CF has grown, with an increasing number of studies highlighting the importance of nocturnal polysomnography or pulse oximetry as routine investigations in CF [15,19,20,21]. Although several studies have explored the effects of ETI on sleep in people with CF, objective assessments using instrumental methods remain limited, and the available evidence is largely based on short-term observations and subjective reports [22,23,24,25,26,27,28]. In this prospective study, we aimed to expand the investigation of ETI therapy by evaluating its effects in a larger and heterogeneous cohort of clinically stable patients, assessing not only its initial benefits but also their long-term persistence.

## 2. Materials and Methods

### 2.1. Patients and Study Design

This was a prospective, single-center, non-profit study conducted between January 2022 and July 2024 at the Pediatric Pulmonology and Cystic Fibrosis Unit of San Marco Hospital, University of Catania, Italy. We included CF patients eligible for ETI, aged over 13 years, to ensure the application of uniform diagnostic criteria for sleep disorders [29]. Only individuals who were clinically stable at the time of follow-up were enrolled. Clinical stability was defined as the absence of any recent deterioration in respiratory symptoms—such as increased dyspnea, cough, or sputum production—together with no new pathological findings on chest examination and no decline in pulmonary function compared with previous assessments. Patients with ongoing or recent pulmonary exacerbations were therefore excluded, and all evaluations were performed during a clinically stable period of at least three months. Additional exclusion criteria included chronic oxygen therapy and previous lung transplantation. Patients using nocturnal non-invasive ventilation (NIV), such as CPAP or BiPAP, were included if they did not require continuous oxygen supplementation and met the stability criteria. Given the small number of NIV users (n = 5) and their clinical stability at baseline, their data were pooled with those of non-NIV users for all analyses.

No formal a priori power calculation was performed, as the study was exploratory and the sample reflected the full eligible population available during the study period.

At baseline (T0), all patients initiated ETI therapy, consisting of two morning tablets containing 75 mg ivacaftor (IVA), 50 mg tezacaftor (TEZA), and 100 mg elexacaftor (ELX), and one evening tablet containing 150 mg IVA. ETI was administered in addition to the patients’ usual standard of care, including airway clearance techniques, inhaled antibiotics or mucolytics, and pancreatic enzyme replacement when indicated.

We collected data on overnight monitoring and pulmonary function tests from each patient at baseline (T0), after one year (T1) and after two years (T2).

The study complied with the Declaration of Helsinki [30], approved by the ethical committee (Ethical Committee n. 278/2021, date of approval 20 December 2021). Informed consent was obtained from all enrolled patients and the parents or legal guardians of the minors involved.

The primary objective of the study was to evaluate longitudinal changes in nocturnal cardiorespiratory polygraphy parameters during ETI therapy compared with baseline, with the aim of determining whether CFTR modulation results in measurable and sustained improvements in sleep-related respiratory function.

### 2.2. Data Collection

Lung function was evaluated by standard spirometry (Cosmed srl, Rome, Italy), according to the European Respiratory Society (ERS) and American Thoracic Society (ATS) criteria [31]. Values of forced expiratory volume in 1 s (FEV1), forced vital capacity (FVC) and forced expiratory flow at 25% (FEF-25), 50% (FEF-50), 75% (FEF-75) were expressed as a percentage of the predicted normal values adjusted for age, sex, height, ethnicity and weight.

Patients underwent nocturnal cardiorespiratory polygraphy at the hospital using VitalNight Plus^®^ (software v.5.19b, VitalAire, Paris, France), while maintaining their usual respiratory support. Each study recorded the following parameters: apnea-hypopnea index (AHI), defined as the number of apnea and/or hypopnea episodes per hour of sleep; oxygen desaturation index (ODI), which represents the number of ≥3% arterial oxygen desaturation events per hour of sleep; mean oxygen saturation (mSpO_2_); time spent with SpO_2_ below 90% (t ≤ 90%); and mean overnight respiratory rate (RR). Polygraphy was scored according to the American Academy of Sleep Medicine (AASM) adult guidelines [29], with AHI ≥ 5.0 events/hour indicating sleep apnea. Desaturation events was defined as ≥3% decreases in SpO_2_. Each recording lasted at least 6 h.

Demographic and anthropometric data—including sex, age, age at ETI initiation, and BMI—were also collected for each participant.

### 2.3. Statistical Analysis

All statistical analyses were performed using SPSS (IBM SPSS Statistic for Windows, version 26.0, IBM Corp., Armonk, NY, USA). Descriptive analyses are presented as medians (25th–75th percentiles) or means ± standard deviation, based on the distribution assessed with the Shapiro–Wilk test. Categorical variables are presented as numbers and percentages. Pre- and post-treatment comparisons were conducted using the Wilcoxon signed-rank test. Effect sizes were calculated using the correlation coefficient r, derived from the formula r = z/√N (Rosenthal, 1991). Interpretation followed Cohen’s (1988) established thresholds: small (≈0.10), medium (≈0.30), and large (≈0.50). Given the exploratory design and limited sample size, more complex longitudinal approaches (such as mixed-effects models) were not applied, as they would not be statistically reliable in this context. Spearman correlation coefficients were computed for T2 variables. The significance level was set at α = 0.05, and *p*-values < 0.05 were considered statistically significant.

## 3. Results

Thirty-five White CF individuals were included (mean age at ETI initiation was 28.6 ± 13.6 SD). Eighteen were female (51.4%) and seventeen male (48.6%). Before treatment, five patients were receiving nocturnal NIV without continuous oxygen supplementation. NIV was discontinued within 12 months of starting ETI due to clinical improvement.

Patient characteristics are summarized in Table S1.

Comparing the sleep study results baseline (T0) to one year after treatment (T1), we observed significant improvement in average oxygen saturation, t ≤ 90%, and overnight respiratory rate (RR). In contrast, no significant changes were detected in ODI or AHI values between T0 and T1. Specifically, the median value of mSpO_2_ at T1 was 97.0% (96.0–98.0) versus 97.0% (94.7–98.0) at T0 (*p* = 0.007, r = 0.45). Similarly, t ≤ 90% decreased significantly from T0 to T1, with the median value decreasing from 0.0 (0.0–0.3) at T0 to 0.0 (0.0–0.0) at T1 (*p* = 0.005, r = 0.47). Mean RR declined from 23.0 (22.0–25.0) at T0 to 22.0 (22.0–23.0) at T1 (*p* = 0.049, r = 0.33).

Spirometry also showed significant improvements after 12 months of therapy, with changes in FEV1, FVC, FEF-25, FEF-50, and FEF-75 being statistically significant (*p* < 0.05). For instance, median FEV1 at T1 was 87.8% (68.8–107.0) versus 68.9% (50.0–94.7) at T0 (*p* < 0.001, r = 0.78). BMI likewise increased significantly at T1. The relevant statistical data are presented in Table 1.

**Figure 1:**
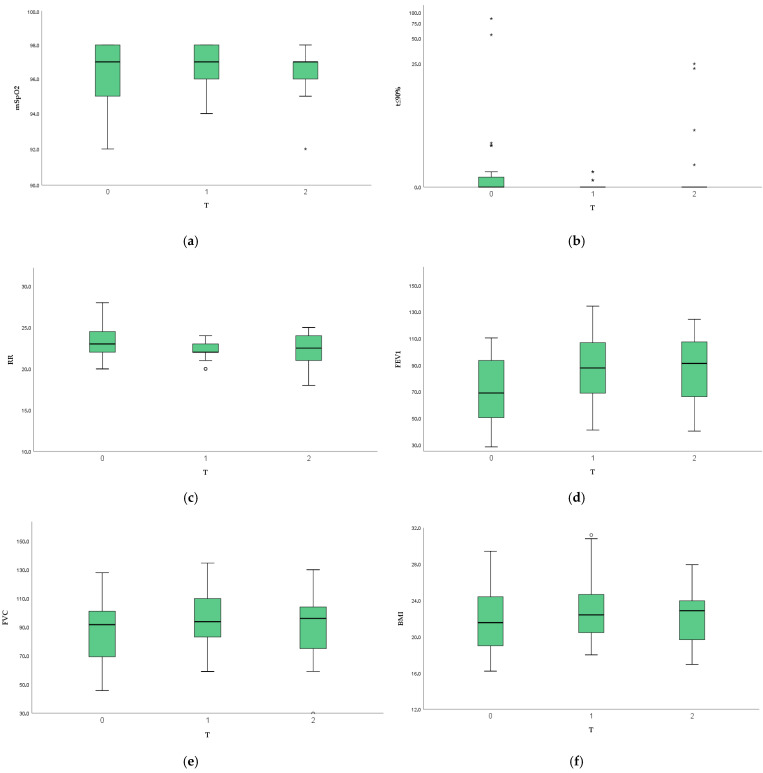
Comparison of mSpO_2_ (**a**), t ≤ 90% (**b**), RR (**c**), FEV1 (**d**), FVC (**e**), BMI (**f**) recorded at T0, T1, and T2. **Legend:** mSpO_2_: mean nocturnal oxygen saturation; t ≤ 90%: time spent with SpO_2_ at or below 90%; RR: mean overnight respiratory rate; FEV_1_: forced expiratory volume in 1 s; FVC: forced vital capacity; BMI: body mass index.

When comparing the results between one year (T1) and two years (T2) of therapy using the same statistical approach, the improvements observed in mSpO_2_, overnight RR, t ≤ 90%, and spirometric parameters were maintained over time, with no further significant changes detected. The results are shown in Table 2. While the comparison between parameters previously mentioned are presented in Figure 1.

**Figure 2:**
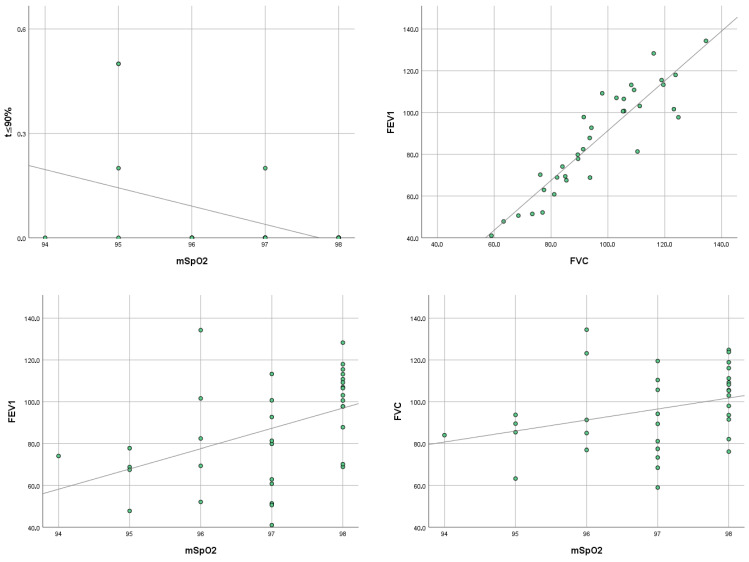
Correlation between mSpO_2_, t ≤ 90%, FEV1 and FVC. **Legend:** mSpO_2_: mean nocturnal oxygen saturation; t ≤ 90%: time spent with SpO_2_ at or below 90%; FEV_1_: forced expiratory volume in 1 s; FVC: forced vital capacity.

We calculated Spearman correlation coefficient for the variables that showed a significant change from baseline to T1. Notably, we found a positive correlation between mSpO_2_ and FEV1 (ρ = 0.515, *p* = 0.002), and between FEV1 and FVC (ρ = 0.894, *p* < 0.001). In contrast to these results, we noted a negative correlation between t ≤ 90% and FEV1 (ρ = 0.404, *p* = 0.016). No significant correlation was found between BMI and any of the other variables. The results are shown in Figure 2, and the full correlation values are provided in Table S2.

## 4. Discussion

Sleep-related respiratory disorders are a significant yet often overlooked comorbidity in patients with CF, even among those who are clinically stable [15]. These disorders may be related to chronic lung disease, nocturnal cough, chronic sinusitis, increasing rates of obesity, and gastrointestinal symptoms [16,17,18,32]. They often arise early in the disease course and negatively affect physical, psychological, and cognitive health [17] underscoring the need for effective screening and treatment strategies to improve patients’ quality of life.

Triple combination therapy has marked a turning point in the natural history of CF. While the efficacy and safety of ETI are well established [33], its effectiveness in managing sleep-related respiratory disorders is still poorly characterized. Our study is the first to evaluate the impact of ETI on nocturnal cardiorespiratory polygraphy parameters with a follow-up period of 24 months. Although polysomnography is considered the gold standard for assessing sleep architecture and arousals, it was not included in this study because our outcomes focused specifically on respiratory sleep parameters, which can be reliably captured using cardiorespiratory polygraphy. Mean oxygen saturation, time with SpO_2_ ≤ 90%, and mean respiratory rate, together with spirometric measurements, showed significant improvement after 12 months, with these gains remaining stable at 24 months of therapy.

An increasing number of studies advocate for the routine use of polysomnography in CF management [19,20,21]. Our results support the integration of sleep studies as a regular component of CF patient evaluations from the early stages of disease.

We observed an increase in nocturnal mSpO_2_ in CF patients after 12 months of ETI therapy. During sleep, especially in the REM phase, episodes of hypoxemia may occur, which can be attributed to hypoventilation or an alteration of the ventilation-perfusion ratio [34].

In the review by Reiter et al. [17], both adults and children with CF experience lower nadir saturation levels and overall SpO_2_ values compared to healthy individuals. Similarly, Silva et al. [35] found decreased nocturnal SpO_2_ levels in a group of children and adolescents with normal or mildly impaired lung function, while Naqvi et al. reported reduced minimal SpO_2_ in the absence of significant respiratory events, even among patients with moderate to severe lung disease [36]. In our cohort, aside from one patient with an average saturation below 92%, no pathological values were identified at baseline. Nevertheless, we showed that ETI therapy significantly improves mean nocturnal oxygen saturation, even when baseline values are within the low-normal range.

Consistent with this improvement, t ≤ 90% also showed a statistically significant increase after 12 months of therapy. However, it is important to note that only two of our patients had a pathological value at T0. We anticipate similar therapeutic success even with a larger sample that includes patients with pathological baseline values. These results emphasize the potential of the therapy to enhance respiratory function during sleep.

In line with our results, a previous study conducted at our center documented the effectiveness of ETI therapy on nocturnal cardiorespiratory polygraphy parameters in nine patients with advanced lung disease and nocturnal ventilation. We concluded that ETI therapy improved nocturnal saturation, both in terms of mSpO_2_ and t ≤ 90%, with benefits evident even three months after initiating therapy, and this improvement was sustained at 12 months [23].

In the present study, however, we found a significant long-term improvement in nocturnal oxygenation, despite involving patients with less severe lung disease. These findings further underscore the importance of incorporating sleep assessment into the routine clinical management of clinically stable CF patients, as they demonstrate that ETI-related benefits extend to earlier stages of the disease.

Furthermore, nocturnal monitoring also revealed a reduction in overnight RR, which had been slightly elevated at baseline. This statistically significant improvement has been documented in the literature [22]. It may be hypothesized that the reduction in respiratory effort following ETI therapy is the result of an overall enhancement in respiratory function and the subsequent increase in average SpO_2_. Conversely, a lower RR decreases oxygen demand and may help maintain more stable nocturnal oxygen saturation. In a previous study, we attributed this improvement to enhanced respiratory muscle strength, as indicated by increases in Maximum Inspiratory Pressure (MIP) and Maximum Expiratory Pressure (MEP) [23].

These findings are consistent with those reported by Dietz-Terjung et al. [24], who also observed improvements in nocturnal respiratory parameters—including AHI, ODI, and respiratory rate—after ETI therapy, although sleep quality, particularly among women, remained suboptimal despite objective gains.

Additionally, our study evaluated another clinically relevant outcome of ETI therapy. At T0, five patients were using nocturnal non-invasive ventilation, and after 12 months of therapy, all were able to discontinue ventilatory support due to overall clinical improvement. None required ventilatory support at T1 or T2. Previous studies have shown similar cases where ETI-induced improvements allowed patients with advanced lung disease to stop ventilation or oxygen therapy, even delaying lung transplantation [23,37].

Although most baseline parameters were within normal limits, we still observed statistically significant improvements following ETI initiation. While the clinical relevance of small changes in already normal parameters may be limited, these findings suggest a favorable trend toward greater respiratory stability that could have implications for long-term health outcomes.

Results regarding the efficacy of ETI on the ODI are inconsistent. While Welsner et al. [22] reported a significant increase in ODI, our previous study [23] in advanced lung disease showed an improvement that was not statistically significant. In our current sample, only one patient presented a pathological ODI value at baseline, which improved after 12 months of ETI therapy. However, overall, we did not observe any statistically significant changes in ODI values in the sample.

In our cohort, AHI and ODI displayed small numerical variations over time, although these did not reach statistical significance. Given the low prevalence of abnormal baseline values, it is unclear whether these trends reflect early treatment-related effects or natural variability. Larger studies including individuals with a greater baseline SRD burden will be needed to determine whether ETI may influence these parameters in a clinically meaningful way.

Our analysis demonstrates an improvement in AHI after ETI therapy, although this change did not reach statistical significance. However, aside from two patients at T0 who were diagnosed with mild obstructive sleep apnea (OSA) (AHI ≥ 5), the nocturnal polygraphy performed prior to therapy did not reveal a pathological AHI in the remainder of the sample. Literature reports a low incidence of nocturnal apneas in adult CF patients [17,22], while OSA is much more prevalent among pediatric CF patients [14] due to chronic sinonasal disease and adeno-tonsillar hypertrophy [38]. In their prospective, observational sleep study, Welsner et al. [22] observed a reduction in AHI and ODI after the initiation of ETI in CF adults, despite normal pre-treatment AHI levels. This was one of the few studies conducted prior to ours that specifically examined the effects of triple combination therapy on nocturnal cardiorespiratory polygraphy parameters; however, the average duration of treatment was only 194 days, which is considerably shorter than that of our participants.

Conversely, Shakkottai et al. [39] were the first to report the effects of CFTR modulator therapy on OSA in children with CF. In contrast to previous findings, this retrospective study observed that children on modulator therapy who underwent overnight monitoring were approximately four times more likely to have OSA compared with those not receiving modulators, despite comparable nutritional status, lung function, and upper airway pathology. The authors therefore highlight the importance of ongoing OSA surveillance in children with CF. However, the study’s limitations include the referral-based population—comprising children with a high clinical suspicion of OSA—and the inclusion of earlier modulator therapies predating the introduction of ETI. Further studies are needed to clarify the true prevalence of OSA in children with CF and to better characterize the modulators’ impact on upper-airway physiology.

In our study, we assessed FEV1, FVC, and FEF-25, FEF-50, FEF-75, all of which demonstrated significant and statistically meaningful improvements following ETI therapy.

Spirometric improvements are well documented in the literature for both adults and children [33,40]. For instance, Sutharsan et al. [40] confirmed an 11.2% increase in FEV1 at 6 months following ETI therapy, which was notably higher than the 1.0% increase observed in the tezacaftor plus ivacaftor group. Similarly, Olivier et al. [41] conducted a retrospective study involving children aged 6 to 17, showing an 11.4% improvement in FEV1 after 3 months of therapy. Savi et al. [42] also reported FEV1 increases of +12.5% at 12 months and +13% at 24 months in 36 patients with advanced lung disease. In contrast, our cohort showed greater FEV1 gains (+27.4% at T1, +32.4% at T2), likely reflecting the inclusion of clinically stable patients with mild to moderate lung disease, consistent with improvements reported in comparable ETI-treated cohorts.

We also assessed BMI, given that adequate nutritional support is essential for optimizing pulmonary function. Before treatment, five patients were underweight (i.e., using the WHO adult criteria, BMI lower than 18.5 kg/m^2^), while the remaining patients had normal or normal-to-low BMI. Consistent with previous reports [43,44,45], all patients gained weight after starting ETI and median BMI increased significantly. Several mechanisms may explain this improvement. This new CFTR modulator increases caloric intake by improving appetite [46] and requiring administration with dietary fats. Additionally, the improvement in respiratory function and the reduction in pulmonary exacerbations may decrease energy expenditure associated with respiratory muscle work and appetite loss, although this has not yet been definitively demonstrated [47,48]. To date, it remains unclear whether ETI is capable of restoring exocrine pancreatic function [49]. However, in our study, fecal elastase was not routinely assessed in patients undergoing pancreatic enzyme therapy. The significant increase in BMI is an encouraging finding; however, as nutritional biomarkers were not collected and nutrition was not a predefined study endpoint, further studies are needed to determine whether this change reflects true nutritional improvement or other ETI-related mechanisms.

Spearman correlation showed a positive correlation at T1 between mSpO_2_ and FEV1 as well as between FEV1 and FVC. The latter correlation was expected, given that FVC is strongly related to FEV1 values. The moderate correlation between mSpO_2_ and FEV1 suggests that improved mSpO_2_ after ETI therapy may depend on FEV1 gains. A negative correlation was also found between t ≤ 90% and FEV1. Previous studies have already investigated the correlation between nocturnal pulse oximetry parameters and respiratory function. Many studies have examined respiratory function parameters (primarily expressed as FEV1, LCI, or daytime saturation) as predictive factors for nocturnal hypoxemia [42,43]. The interplay between these parameters suggests that improvements in spirometric measurements not only directly affect daytime respiratory function but may also contribute to better nocturnal oxygen saturation. This strengthens the idea that the benefits of ETI therapy extend beyond daytime pulmonary function, contributing to improved nocturnal respiratory parameters and better management of sleep-related breathing disorders. These functional correlations are consistent with imaging evidence showing that ETI leads to structural lung improvements, including reductions in mucus plugging, airway wall thickening, air trapping, and inflammatory changes. By enhancing airway patency and ventilation homogeneity, these anatomical modifications provide a common mechanistic basis for the parallel improvements observed in both spirometric indices and nocturnal oxygenation. Thus, pulmonary function tests and SRD changes should be interpreted as concomitant manifestations of the same ETI-induced structural recovery, rather than as a direct causal relationship between daytime and nocturnal respiratory function.

Ultimately, BMI improvements did not correlate with the other variables. Thus, the weight gain observed in CF patients after treatment appeared to be independently associated with improvements in both spirometric and polygraphic parameters, which may be interpreted as a general improvement in health among these individuals.

In our study, we compared outcomes after 12 and 24 months of ETI therapy. The significant clinical improvements observed from baseline to 12 months, including both spirometric parameters and nocturnal cardiorespiratory polygraphy measures, suggesting a broader overall enhancement in health status in these individuals.

To our knowledge, all studies on the efficacy of ETI therapy have reported improvements in the measured parameters within the first year of treatment [23,41]. In particular, our group and Olivier et al. [23,41] previously demonstrated a positive impact on pulmonary function tests after 3 months of ETI therapy, which was sustained at the 12 and 6-month follow-up, respectively. Our study is among the few to demonstrate sustained clinical benefits of ETI therapy over a 24-month period, confirming that the improvements observed at one year persist for another 12 months. This supports the conclusion that the clinical improvement achieved after one year of ETI therapy are stable and enduring throughout an additional 12 months of treatment. Overall, our study adds valuable data to the existing evidence, showing that the therapeutic benefits of ETI therapy are not transient but rather sustained over a longer period.

This study has some limitations. The absence of a predefined power calculation and the modest sample size, particularly given the low prevalence of abnormal baseline sleep parameters, may have limited the ability to detect significant changes in AHI and ODI. Although we did not use validated questionnaires to assess subjective sleep quality—which could have added insight into patients’ perceptions—the objective improvements in nocturnal respiratory parameters support the relevance of instrumental sleep studies in monitoring ETI therapy. Despite the limited sample size, the consistency of our findings highlights the need for validation in larger, preferably multicenter, studies. The use of race-based spirometric equations is another limitation to be addressed in future analyses. Further research should include subjective assessments, broader demographic representation, control groups, and extended follow-up beyond 24 months to better define the clinical significance and long-term impact of ETI on nocturnal respiratory health.

## 5. Conclusions

Current evidence on sleep disturbances in individuals with cystic fibrosis—particularly those receiving ETI therapy—remains limited. This study provides additional insight into the effects of ETI therapy on nocturnal cardiorespiratory polygraphy parameters in people with CF over a 24-month period. Our findings indicate that ETI therapy significantly enhances mean oxygen saturation levels, reduces the time spent with oxygen saturation below 90%, and lowers mean respiratory rate after 12 months. These improvements likely reflect the same ETI-induced structural and physiological benefits that also drive the spirometric gains. Importantly, the persistence of these improvements over 24 months indicates that the benefits of ETI therapy are sustained in the long term.

These findings emphasize the importance of incorporating the evaluation of cardiorespiratory parameters through sleep studies into the clinical management of people with CF, beginning in the early stages of the disease. For this reason, nocturnal respiratory monitoring should be considered an integral component of routine clinical follow-up in people with CF, including those who appear clinically stable, to ensure early detection of sleep-related disturbances and a more comprehensive assessment of ETI’s long-term benefits.

## Figures and Tables

**Table 1:** Comparison between T0 and T1.

	T0	T1	z	*p*	r
AHI	3.30 (1.00–3.70)	2.20 (1.80–3.80)	−0.080	0.936	0.001
ODI	2.50 (1.20–3.32)	3.20 (1.60–4.20)	−1.132	0.258	0.19
mSpO_2_	97.0 (94.7–98.0)	97.0 (96.0–98.0)	−2.679	0.007	0.45
t ≤ 90%	0.00 (0.00–0.32)	0.00 (0.00–0.00)	−2.805	0.005	0.47
overnight RR	23.0 (22.0–25.0)	22.0 (22.0–23.0)	−1.973	0.049	0.33
FEV1	68.9 (50.0–94.7)	87.8 (68.8–107.0)	−4.619	<0.001	0.78
FVC	91.6 (66.0–101.2)	93.7 (82.1–110.4)	−4.234	<0.001	0.71
FEF-25	58.5 (38.9–93.3)	78.6 (46.3–104.5)	−3.861	<0.001	0.65
FEF-50	45.2 (21.8–68.9)	60.5 (35.6–93.8)	−4.210	<0.001	0.71
FEF-75	30.6 (14.3–48.4)	36.4 (24.1–67.1)	−3.669	<0.001	0.11
BMI	21.5 (19.0–24.4)	22.4 (20.1–24.7)	−3.906	<0.001	0.66

**Legend:** mSpO_2_: mean nocturnal oxygen saturation; t ≤ 90%: time spent with SpO_2_ at or below 90%; RR: mean overnight respiratory rate; FEV_1_: forced expiratory volume in 1 s; FVC: forced vital capacity; BMI: body mass index; Data are expressed as median and interquartile range. Effect sizes are reported as r, calculated from the z-value (r = z/√N). Significance level was set at *p* < 0.05.

**Table 2:** Comparison between T1 and T2.

	T1	T2	z	*p*	r
AHI	2.20 (1.80–3.80)	2.20 (0.80–3.40)	−1.400	0.161	0.24
ODI	3.20 (1.60–4.20)	3.00 (2.27–4.30)	−0.081	0.936	0.01
mSpO_2_	97.0 (96.0–98.0)	96.8 (96.0–97.0)	−1.234	0.217	0.21
t ≤ 90%	0.00 (0.00–0.00)	0.00 (0.00–0.00)	−1.572	0.116	0.27
overnight RR	22.0 (22.0–23.0)	22.5 (20.7–24.0)	−0.467	0.641	0.08
FEV1	87.8 (68.8–107.0)	91.1 (65.5–107.9)	−0.483	0.629	0.08
FVC	93.7 (82.1–110.4)	96.0 (74.6–104.8)	−1.549	0.121	0.26
FEF-25	78.6 (46.3–104.5)	89.5 (36.4–106.0)	−0.089	0.929	0.02
FEF-50	60.5 (35.6–93.8)	71.1 (20.9–94.6)	−0.597	0.551	0.10
FEF-75	36.4 (24.1–67.1)	56.0 (23.0–87.4)	−1.511	0.131	0.26
BMI	22.4 (20.1–24.7)	22.4 (20.1–24.7)	−0.704	0.481	0.12

**Legend:** mSpO_2_: mean nocturnal oxygen saturation; t ≤ 90%: time spent with SpO_2_ at or below 90%; RR: mean overnight respiratory rate; FEV_1_: forced expiratory volume in 1 s; FVC: forced vital capacity; BMI: body mass index; Data are expressed as median and interquartile range. Effect sizes are reported as r, calculated from the z-value (r = z/√N). Significance level was set at *p* < 0.05.

## Data Availability

The data presented in this study are available on request from the corresponding author due to privacy.

## Supplementary Materials

The following supporting information can be downloaded at https://www.mdpi.com/article/10.3390/life15121942/s1. Table S1: Characteristics of patients at T0. Table S2: Correlation between mSpO2, t ≤ 90%, FEV1, FVC, and BMI.
