# Supplementary material for: Long-Term Effects of Elexacaftor/Tezacaftor/Ivacaftor on Nocturnal Cardiorespiratory Polygraphy Parameters in Patients with Cystic Fibrosis: A Prospective Study
Source: Life (Basel). 2025 Dec 18;15(12):1942. doi: 10.3390/life15121942 (PMC12735109; doi:10.3390/life15121942)
Supplement: Supplementary file 1 [file life-15-01942-s001.zip › life-3992505-supplementary.pdf]

Table S1. Characteristics of patients at T0.

**N°**	**35**
**Female/Male (N°; %)**	18; 51.4% / 17; 48.6%
**Age (y)**	28.6 ± 13.6*
**BMI (kg/m²)**	21.6 (19.0-24.4)
**AHI (N°/h)**	3.3 (1.0-3.7)
**ODI**	2.5 (1.2-3.3)
**mSpO2 (%)**	97.0 (94.7-98.0)
**T ≤ 90%**	0.0 (0.0-0.3)
**overnight RR (bpm)**	23.0 (22.0-25.0)
**FEV1 (%)**	68.9 (50.0-94.7)
**FVC (%)**	91.6 (66.0-101.2)
**FEF-25 (%)**	58.5 (38.9-93.3)
**FEF-50 (%)**	45.2 (21.8-68.9)
**FEF-75 (%)**	1.6 (14.3-48.4)

* Described as mean ± standard deviation.

Table S2. Correlation between mSpO2, t≤90%, FEV1, FVC, and BMI.

		**mSpO2**	**t≤90%**	**FEV1**	**FVC**	**BMI**
**mSpO2**	ϱ	1.000	-0.438	0.515	0.359	-0.060
	p	/	0.008	0.002	0.034	0.731
**t≤90%**	ϱ	0.438	1.000	-0.404	-0.282	-0.059
	p	0.008	/	0.016	0.100	-0.735
**FEV1**	ϱ	0.515	-0.404	1.000	0.894	0.175
	p	0.002	0.016	/	<0.001	0.314
**FVC**	ϱ	0.359	-0.282	0.894	1.000	0.099
	p	0.034	0.100	<0.001	/	0.570
**BMI**	ϱ	-0.060	-0.059	0.175	0.099	1.000
	p	0.731	0.735	0.314	0.570	/

ϱ: Spearman correlation coefficient.
